# miR-146a promotes growth of osteosarcoma cells by targeting ZNRF3/GSK-3β/β-catenin signaling pathway

**DOI:** 10.18632/oncotarget.19395

**Published:** 2017-07-19

**Authors:** Chun Zhou, Chang-Qing Jiang, Zhen Zong, Jia-Chen Lin, Li-Feng Lao

**Affiliations:** ^1^ Department of Orthopaedic Surgery, Renji Hospital, School of Medicine, Shanghai Jiao Tong University, Shanghai, China

**Keywords:** miR-146a-5p, growth, ZNRF3, osteosarcoma, recurrence

## Abstract

MicroRNA-146a-5p (miR-146a) functions as a tumor suppressor or oncogene involved in multiple biological processes. But, the underlying molecular mechanisms by which miR-146a contributes to osteosarcoma (OS) remain unclear. The correlation of miR-146a expression with clinicopathologic characteristics and prognosis of OS patients was analyzed by Kaplan-Meier and Cox regression analysis. Cell growth *in vitro* and *in vivo* was assessed by MTT, cell colony formation and animal models. The target of miR-146a was identified by bioinformatics software and gene luciferase reporter. As a result, miR-146a expression was substantially elevated in OS tissues and was positively associated with the tumor size (*P*=0.001) and recurrence (*P*=0.027) of OS patients. Moreover, knockdown of miR-146a suppressed cell proliferation and colony formation *in vitro* and *in vivo*. In addition, zinc and ring finger 3 (ZNRF3) was identified as a direct target of miR-146a in OS cells, and was negatively correlated with miR-146a expression in OS tissues. Overexpression of ZNRF3 inhibited cell growth and rescued the tumor-promoting role of miR-146a via inhibition of GSK-3β/β-catenin signaling pathway. Taken together, miR-146a may function as an oncogene in OS cells by targeting ZNRF3/GSK-3β/β-catenin signaling pathway, and represent a promising biomarker for OS patients.

## INTRODUCTION

Osteosarcoma (OS) is considered to be an aggressive bone neoplasm and occurs mainly in children and young adults [1]. Although the great progress has been made in surgical treatment combined with multidrug chemotherapy, the five-year survival rate and recurrence risk of OS patients have not been significantly improved due to the tumor local relapse and metastases [2]. Many molecular alterations in critical signal transduction pathways contribute to the OS pathogenesis via promoting the proliferation and metastasis [3]. Herein, identifying the key molecular targets involved in OS progression will be highlighted for diagnosis and therapy of OS patients.

MicroRNAs (miRNAs) as noncoding RNAs have participated in multiple biological and pathogenesis of diseases by regulating gene expression. Dysregulation of miRNAs as oncogenes or tumor suppressors is involved in multiple steps of the oncogenic process [4]. On the one hand, miR-146a expression is found upregulated in gastric cancer [5], breast cancer [6], premalignant thymocytes [7] and thyroid cancer [8], and the rs2910164 polymorphism in miR-146a is related with the incidence of melanoma [9]. Ectopic expression of miR-146a results in cell proliferation [8, 10], migration [10], invasion and metastasis [11] in some cancers. Moreover, some key pathways mediate miR-146a promoting tumor progression. miR-146a facilitates growth and development of melanoma by activation of Notch signaling [12], reduces cell apoptosis in acute promyelocytic leukemia by Smad4 signaling [13] and enhances tumorigenic potential of breast cancer [14]. These data indicate that miR-146 may function as an oncogene involved in tumor progression.

On the other hand, miR-146a expression is decreased in various cancers, such as NK/T cell lymphoma [15], gastric cancer [16], castration-resistant prostate cancer [17], head and neck squamous cell carcinoma [18], hepatocellular carcinoma [19], penile squamous cell carcinoma [20] and non-small cell lung cancer [21]. Enforced expression of miR-146a suppresses cell proliferation and invasion [15-19, 21-24) and angiogenesis via targeting EGFR [17], RAF6 [19], CCND1/2 [21], Notch1 [22] or Rac1 [24], induces cell apoptosis [15, 16, 23] and enhanced chemo-sensitivity [15]. Therefore, miR-146 may function as a tumor suppressor for cancer therapy.

Despite the bi-directional role of miR-146a in cancers, its function and underlying regulation mechanism remain unclear in OS. In the present study, we found that miR-146a expression was increased in OS tissues and was positively correlated with the tumor size and high recurrence rate of OS patients. Knockdown of miR-146a suppressed cell growth *in vitro* and *in vivo*. In addition, ZNRF3 was identified as a direct target of miR-146a in OS cells, and had a reverse correlation with miR-146a expression in OS tissues. Overexpression of ZNRF3 inhibited cell growth and attenuated the tumor-promoting effects of miR-146a via inhibition of GSK-3β/β-catenin signaling, indicating that miR-146a may function as a tumor suppressor and represent a potential biomarker for OS patients.

## RESULT

### miR-146a expression was upregulated in human OS tissues

**Figure 1:**
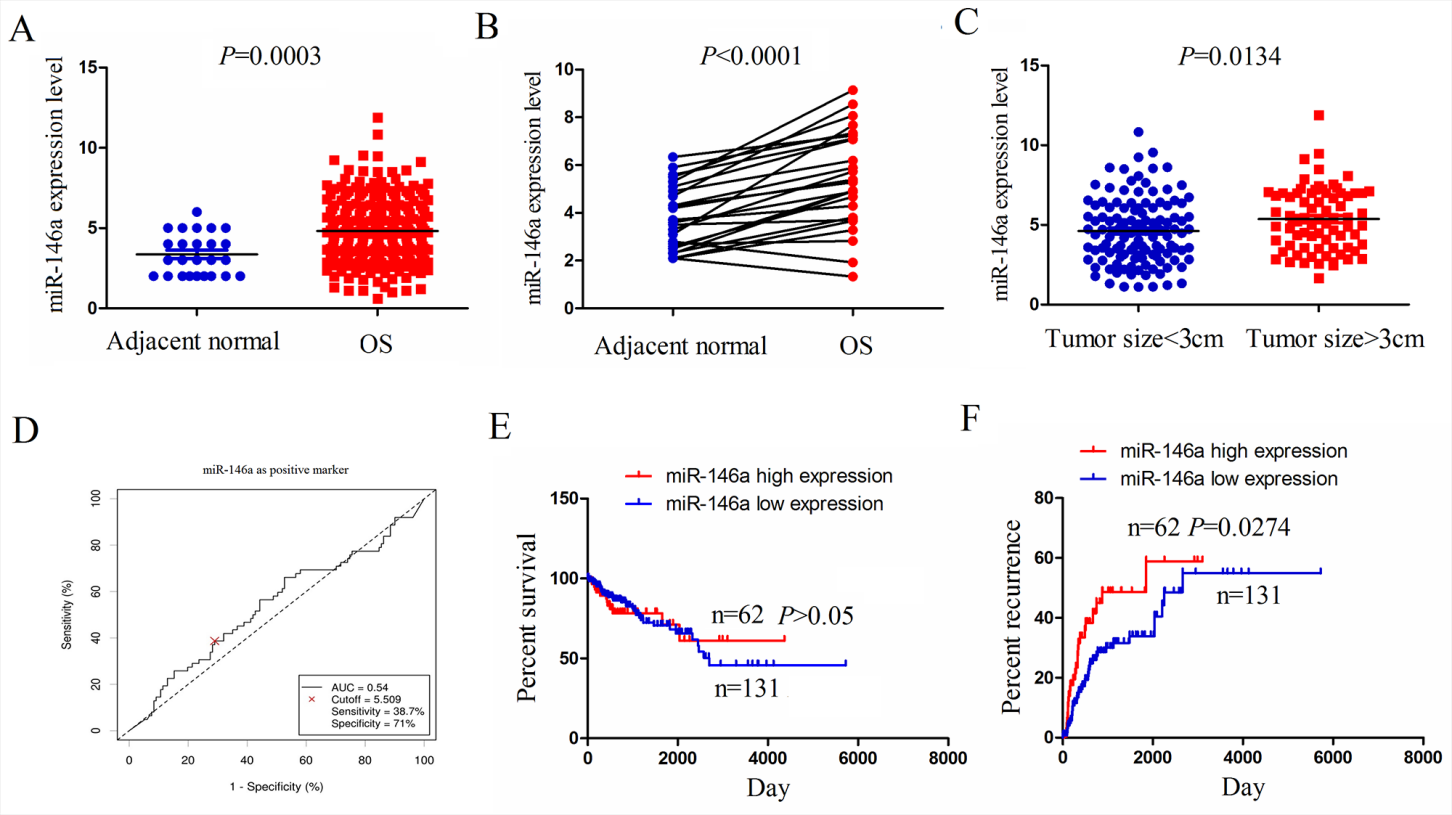
The expression of miR-146a was upregulated in OS tissues and associated with tumor recurrence **(A)** The expression of miR-146a in 237 OS patient samples and 25 normal tissues shown by a 2015 TCGA data set. **(B)** TCGA data analysis of the expression of miR-146a in paired OS tissues (n=25). **(C)** The expression level of miR-146a in OS patients with tumor size≥8cm (n-74) and tumor size<8cm (n=119). **(D)** The cutoff value of miR-146a was determined by the patient survival time, status and miR-146 expression level. **(E)** The correlation of miR-146a high expression or low expression with the overall survival rate of OS patients. **(F)** The correlation of miR-146a high expression or low expression with the tumor recurrence rate of OS patients.

To confirm the expression level of miR-146a in human OS tissues, we used the 2015 The cancer Genome Atlas (TCGA) gene sequencing data (https://genome-cancer.ucsc.edu/) to examine miR-146a expression in OS tissues, indicating that miR-146a was highly expressed in OS tissues (n=237) compared to the adjacent normal tissues (n=25) (Figure 1A). miR-146a expression was also upregulated in OS tissues (n=25) compared with the pair-matched normal tissues (n-25) (Figure 1B). To uncover the link between miR-146a expression and tumor growth in OS tissues, we detected the expression of miR-146a in OS with tumor size ≥3cm (n=74) or tumor size <3cm (n=119), indicating that miR-146a expression was substantially upregulated in OS with tumor size ≥3cm compared with those with tumor size <3cm (Figure 1C).

### The correlation of miR-146a expression with clinicopathologic features and prognosis of OS patients

We further analysed the association of miR-146a expression with clinicopathologic characteristics and prognosis of OS patients. As indicated in Table 1, miR-146a expression was positively associated with tumor size (*P*=0.004), but had no correlation with age, gender, lymph node infiltration and distant metastasis in OS patients (each *P*>0.05). According to the overall survival time, survival status and miR-146a expression level, we used the cutoff finder (http://molpath.charite.de/cutoff/load.jsp) to define the high expression level (cutoff ≥ 5.509) or low expression level (cutoff <5.509) of miR-146a in OS tissues (Figure 1D). We then analyzed the association between miR-146a high expression and overall survival and recurrence in OS patients by using Kaplan-Meier and Multivariate analysis, indicating that miR-146a expression was not an independent prognostic factor for overall survival (Supplementary Table 1) and tumor recurrence (Supplementary Table 2) in OS patients. Overall survival curve demonstrated that OS patients with miR-146a high expression had no difference in comparison with the patients with miR-146a low expression (Figure 1E), but the recurrence curve showed that the patients with miR-146a high expression had higher tumor recurrence rate than the patients with miR-146a low expression (Figure 1F).

**Table 1:** The correlation of miR-146a expression with clinicopathological factors of OS patients

Variables	Cases (n)	miR-146a low	Expression high	*P* value
Total	193	131	62	
Age (years)				
≥20	83	61	22	
< 20	110	70	40	0.148
Gender				
Male	89	57	32	
Female	104	74	30	0.293
Tumor size (cm)				
≥3	74	41	33	
<3	119	90	29	0.004
Lymph node infiltration				
No	97	69	28	
Yes	96	62	34	0.331
Distant metastases				
No	168	115	53	
Yes	25	16	9	0.657

### The effects of miR-146a on OS cell growth

**Figure 2:**
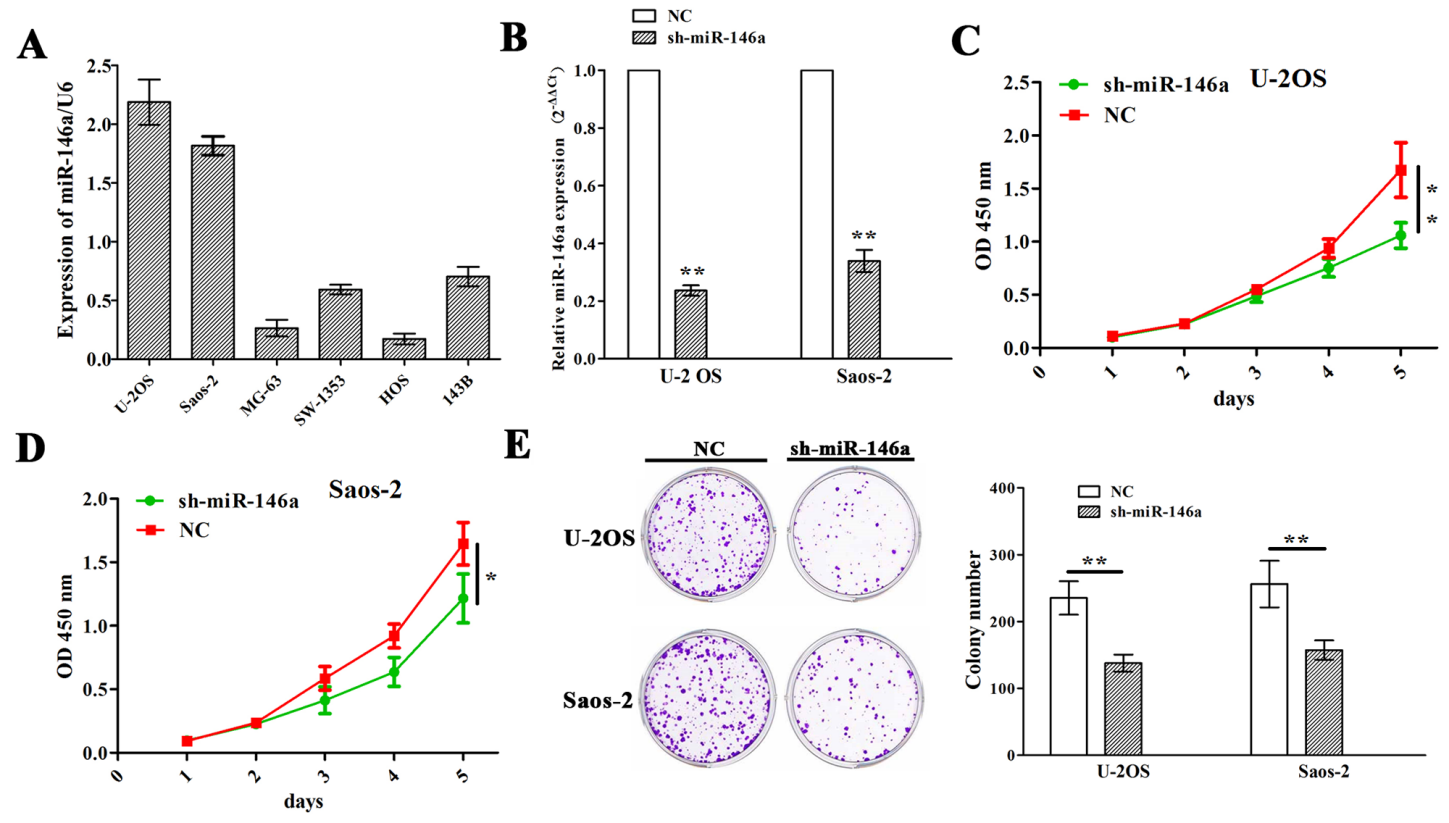
Knockdown of miR-146a inhibited OS cell proliferation and colony formation **(A)** qRT-PCR analysis of the expression levels of miR-146a in multiple OS cell lines. **(B)** qRT-PCR analysis of the knockdown efficiency of sh-miR-146a in U-2OS and Saos-2 cells. **(C** and **D)** The effects of miR-146a knockdown on cell proliferation of U-2OS and Saos-2 cells indicated by MTT assay. **(E)** The effects of miR-146a knockdown on cell colony formation of U-2OS and Saos-2 cells. * *P*<0.05, **P<0.01.

Having verified the positive correlation of miR-146a expression with tumor size of OS patients (Figure 1C and Table 1), we then explored the functions of miR-146a in OS growth. We first examined the expression levels of miR-146a in different OS cell lines (U-2 OS, Saos-2, MG-63, SW-1353, HOS and 143B), and found that miR-146a was highly expressed in U-2 OS and Saos-2 cell lines but lowly expressed in MG-63 and HOS cell lines (Figure 2A). Then, miR-146a shRNA vector was transfect intro U-2 OS and Saos-2 cell lines with miR-146a high expression. After transfection for 48h, miR-146a expression levels were determined by qRT-PCR, indicating a decreased miR-146a expression in sh-miR-146a group compared to NC group (Figure 2B). Then, MTT and colony formation assays showed that knockdown of miR-146a significantly reduced cell proliferation activity (Figure 2C and 2D) and colony formation (Figure 2E) in OS cells.

**Figure 3:**
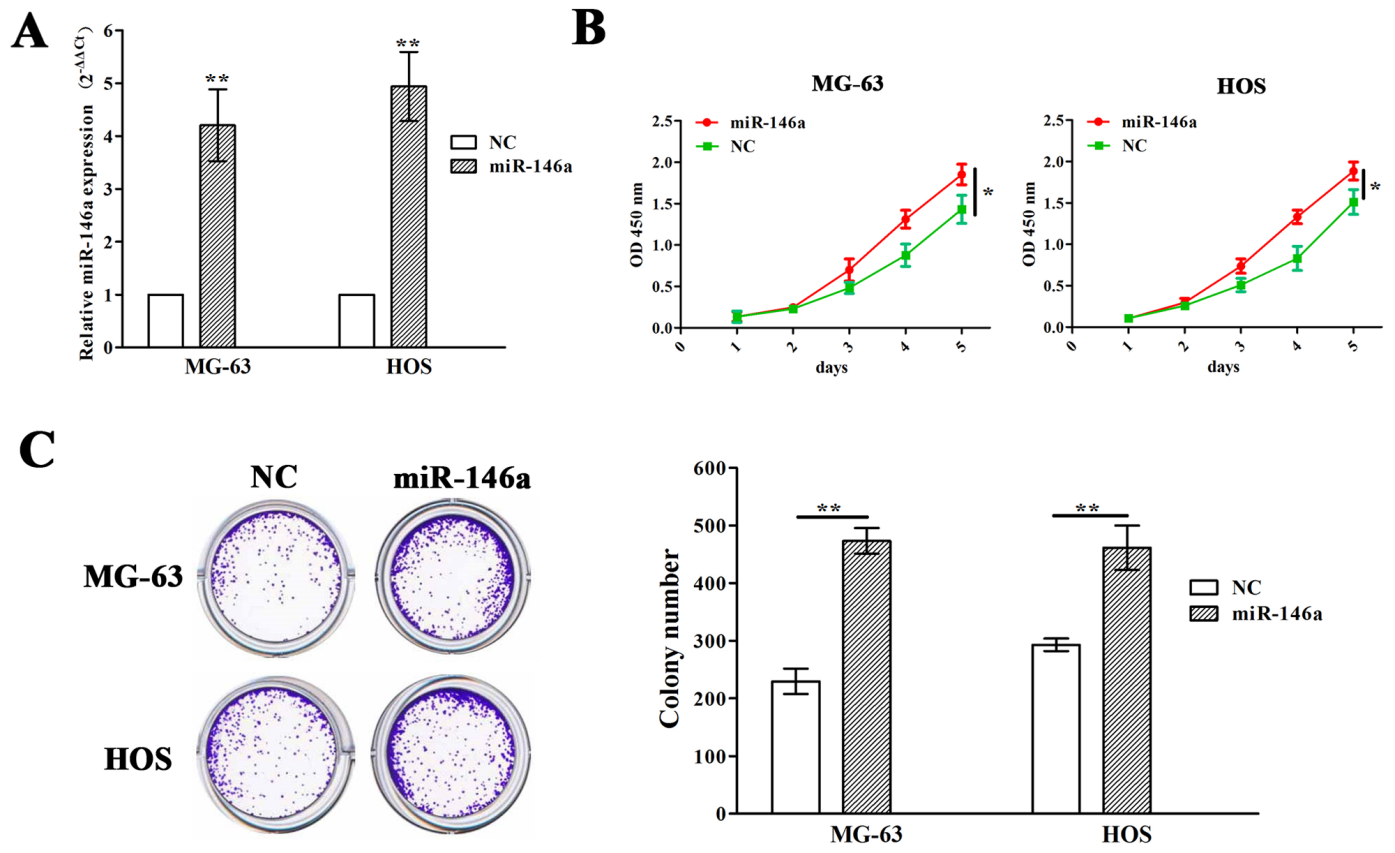
Ectopic expression of miR-146a promoted OS cell proliferation and colony formation **(A)** qRT-PCR analysis of the transfection efficiency of lentivirus mediated miR-146a in MG-63 and HOS cells. **(B)** The effects of miR-146a overexpression on cell proliferation of MG-63 and HOS cells indicated by MTT assay. **(C)** The effects of miR-146a overexpression on cell colony formation of MG-63 and HOS cells. * *P*<0.05, **P<0.01.

Meanwhile, miR-146a overexpression vector was used to transfect into MG-63 and HOS cell lines with miR-146a low expression. After transfection for 48h, miR-146a expression levels were markedly increased in miR-146a group compared with NC group (Figure 3A). MTT and colony formation assays showed that overexpression of miR-146a promoted cell growth of MG-63 and HOS cells (Figure 3B and 3C).

### ZNRF3 was a target gene of miR-146a in OS cells

**Figure 4:**
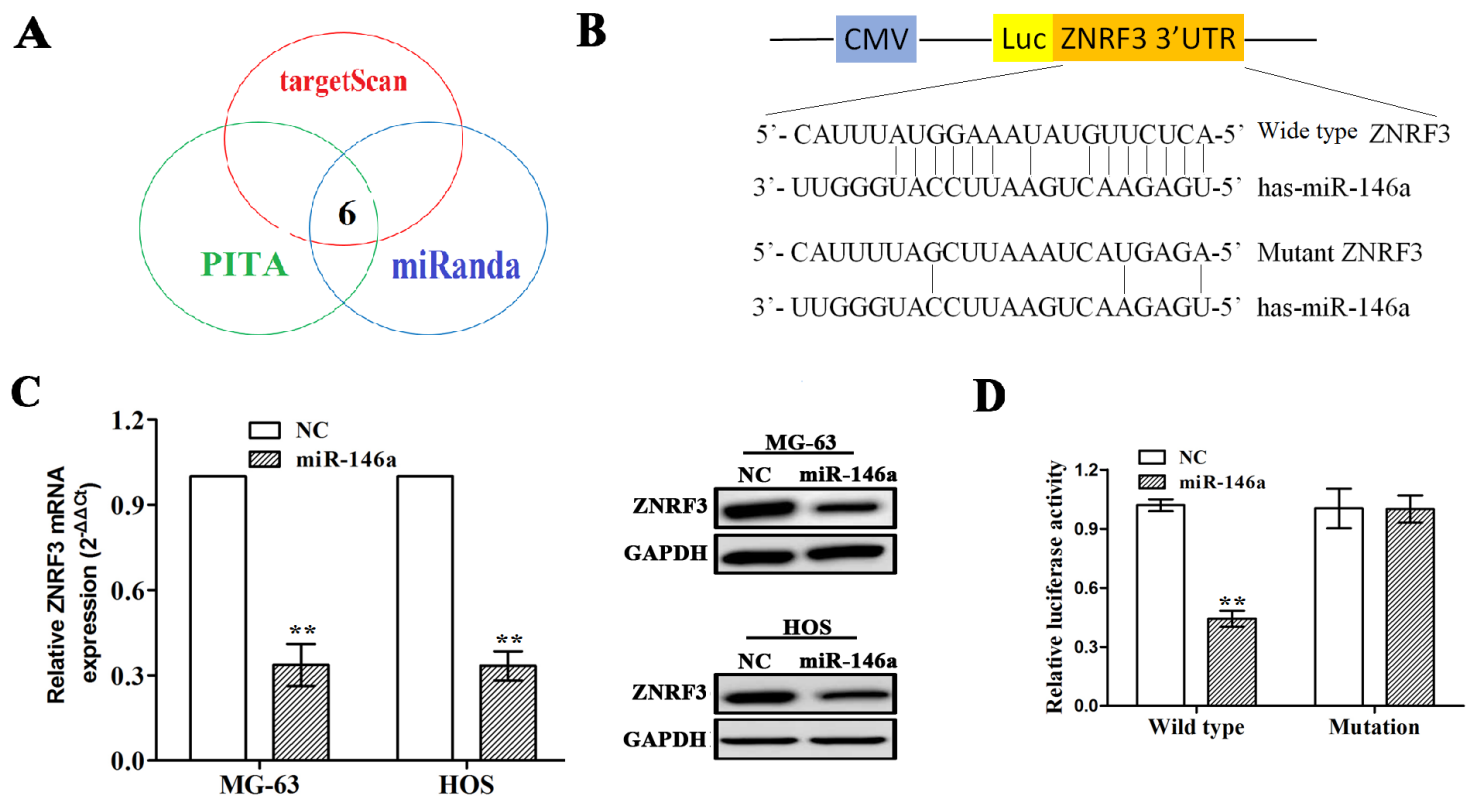
ZNRF3 gene was identified as a direct target of miR-146a **(A)** 6 target genes of miR-146a were identified by miRNA forecasting software. **(B)** Diagrams demonstrated the miR-146a putative binding sites and corresponding mutant sites of ZNRF3. **(C)** The expression levels of ZNRF3 were examined after transfection with miR-146a by qRT-PCR and Western blotting assays in MG-63 and HOS cells. **(D)** Luciferase activity of ZNRF3 (wild type or mutation) was evaluated after miR-146a transfection into MG-63 and HOS cells. **P<0.01.

To confirm the molecular mechanism by which miR-146a regulates cell growth in OS cells, we identified the potential target genes of miR-146a by using prediction websites (http://starbase.sysu.edu.cn/targetSite.phpfor). As shown in Figure 4A, under the stringent conditions including very high stringency (≥5), cancer types (≥5) and prediction software (≥3), six target genes were identified to have the potential to bind with miR-146a, of which zinc and ring finger 3 (ZNRF3) was considered as the most suitable candidate target gene of the miR-146a due to its highest binding force (Supplementary Table 3). To further verify whether miR-146a directly binds to the 3’ UTR of ZNRF3, the wild type 3’ UTR or the mutant 3’ UTR target sequences of ZNRF3 (Figure 4B) were cloned into the luciferase reporter vector in MG-63 and HOS cells. It was demonstrated that miR-146a decreased the expression levels of ZNRF3 in OS cells (Figure 4C). Moreover, miR-146a overexpression lowered the luciferase activity of wild type 3` UTR of ZNRF3 in OS cells, but had no effect on that of mutation 3` UTR of ZNRF3 (Figure 4D).

### ZNRF3 suppressed cell proliferation and colony formation of OS cells

**Figure 5:**
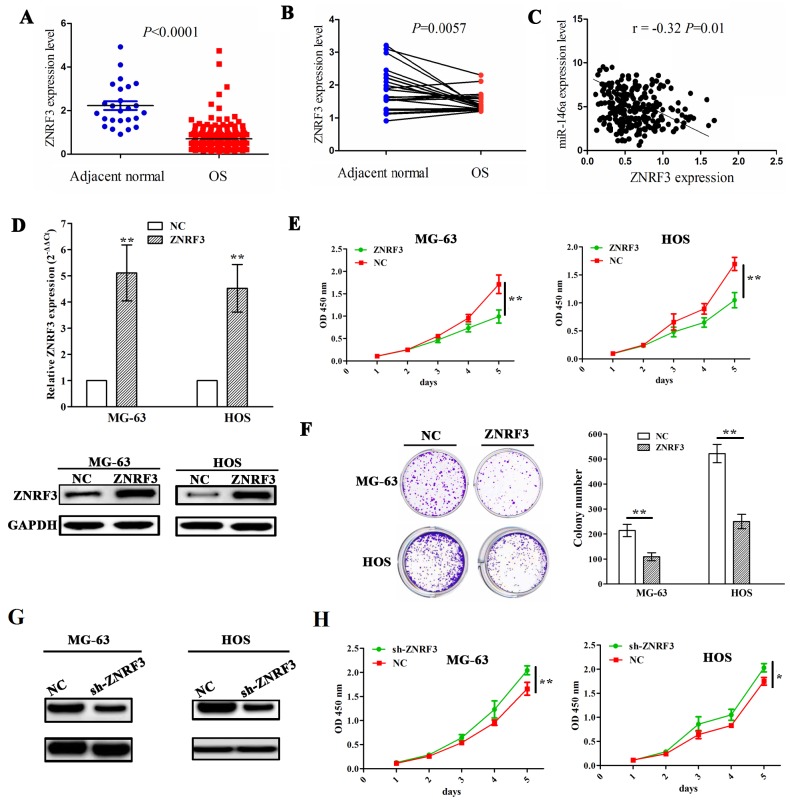
Enforced expression of ZNRF3 inhibited OS cell proliferation and colony formation **(A)** The expression of ZNRF3 in OS tissues (n=237) and the adjacent normal tissues (n=25) shown by TCGA data. **(B)** The expression of ZNRF3 in paired OS tissues (n=25). **(C)** Thecorrelation analysis showed the association of miR-146a expression with ZNRF3 in OS patients. **(D)** The transfection efficiency of ZNRF3 overexpression was determined by qPCR and western blot assays in MG-63 and HOS cells. **(E)** The effects of ZNRF3 overexpression on cell proliferation of MG-63 and HOS cells by MTT assay. **(F)** The effects of ZNRF3 overexpression on cell colony formation of MG-63 and HOS cells. **(G)** Western blot analysis of the transfection efficiency of ZNRF3 knockdown in MG-63 and HOS cells transfected with sh-ZNRF3 and NC. **(H)** The effects of ZNRF3 knockdown on cell proliferation of MG-63 and HOS cells by MTT assay. * *P*<0.05, **P<0.01.

To demonstrate the association of miR-146a expression with ZNRF3 in OS tissues, we first measured the expression level of ZNRF3 in OS tissues (n=237) and adjacent normal tissues (n=25) by TCGA data, which uncovered that ZNRF3 expression was substantially downregulated in OS tissues (Figure 5A). ZNRF3 expression was also decreased in OS tissues compared with pair-matched normal tissues (Figure 5B). The correlation analysis showed the negative correlation of miR-146a with ZNRF3 expression in OS tissues (Figure 5C). Furthermore, we constructed the ZNRF3 overexpression stably transfected MG-63 and HOS cell lines, and confirmed its transfection efficiency by qRT-PCR and western blotting assays (Figure 5D). Then, ectopic expression of ZNRF3 significantly repressed cell proliferation (Figure 5E) and colony formation (Figure 5F), but knockdown of ZNRF3 by sh-ZNRF3 (Figure 5G) promoted cell proliferation in OS cells (Figure 5H).

### ZNRF3 rescued the proliferation-promoting effects of miR-146a via GSK-3β/β-catenin pathway

**Figure 6:**
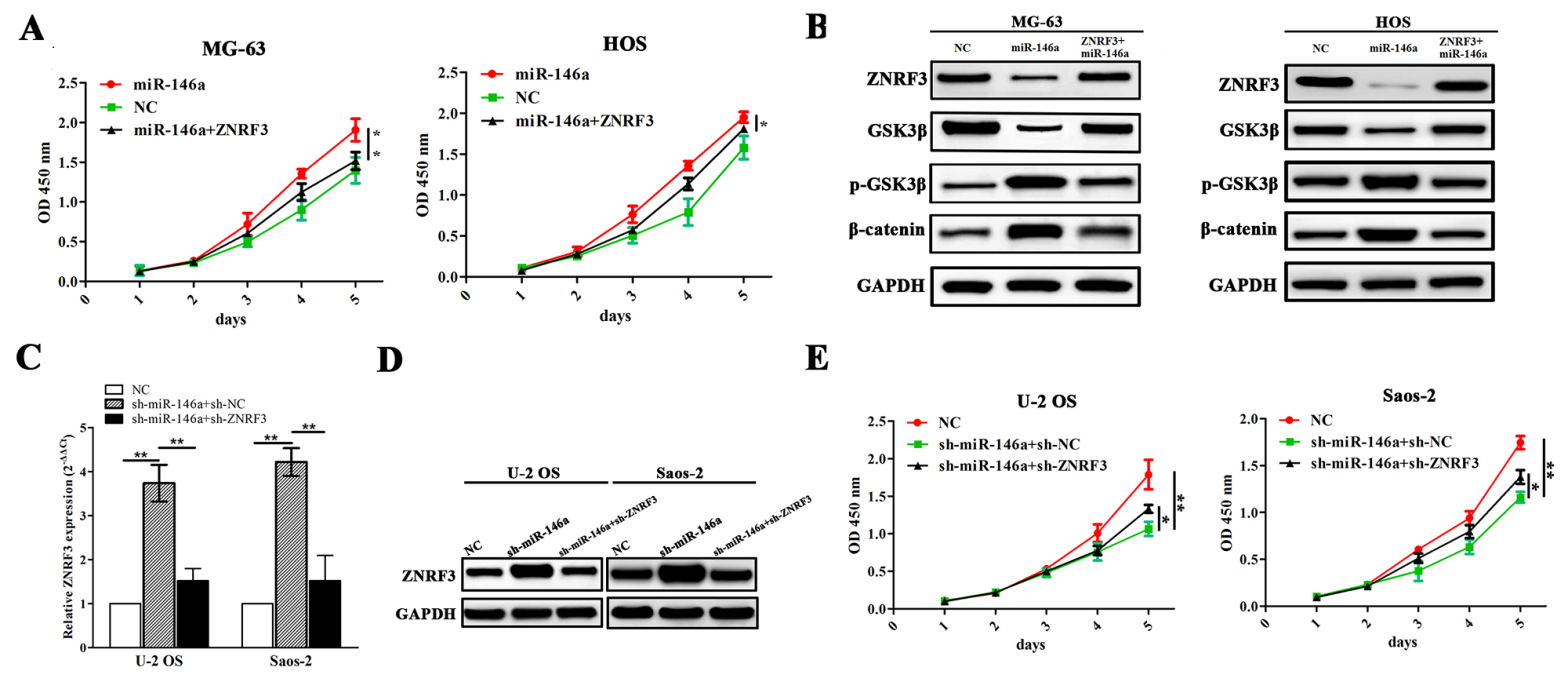
ZNRF3 overexpression rescued tumor proliferation-promoting effects by miR-146a **(A)** The effects of ZNRF3 overexpression on cell proliferation in miR-146a-transfected MG-63 and HOS cells indicated by MTT assay. **(B)** The effects of ZNRF3 overexpression on the protein expression of GSK-3β/β-catenin pathway in miR-146a-transfected OS cells indicated by western blotting. **(C, D)** Real-time PCR and western blot analysis of the expression levels of ZNRF3 in sh-ZNRF3 and sh-miR-146a co-transfected U-2 OS and Saos-2 cells. (**E**) MTT analysis of cell proliferative activities of U-2 OS and Saos-2 cells co-transfected by sh-ZNRF3 and sh-miR-146a. *P<0.05, **P<0.01.

To uncover the molecular mechanism by which ZNRF3 rescues the proliferation-promoting effects induced by miR-146a, we co-transfected ZNRF3 and miR-146a overexpression vectors into MG-63 and HOS cells, indicating that ZNRF3 overexpression reduced miR-146a-induced cell proliferation (Figure 6A). Moreover, the expression levels of GSK-3β and β-catenin as potential downstream factors of ZNRF3 were detected by Western blot, indicating that miR-146a increased the activity of GSK-3β/β-catenin pathway, but this effect was reversed by ZNRF3 in OS cells (Figure 6B). Then, we co-transfected sh-ZNRF3 and miR-146a knockdown vectors into OS U2-OS and Saos-2 cells, and found that knockdown of ZNRF3 attenuated the effects of sh-miR-146a induced increased expression of ZNRF3 (Figure 6C and 6D) and decreased proliferation of OS cells (Figure 6E).

### miR-146a knockdown inhibited xenograft tumor growth

**Figure 7:**
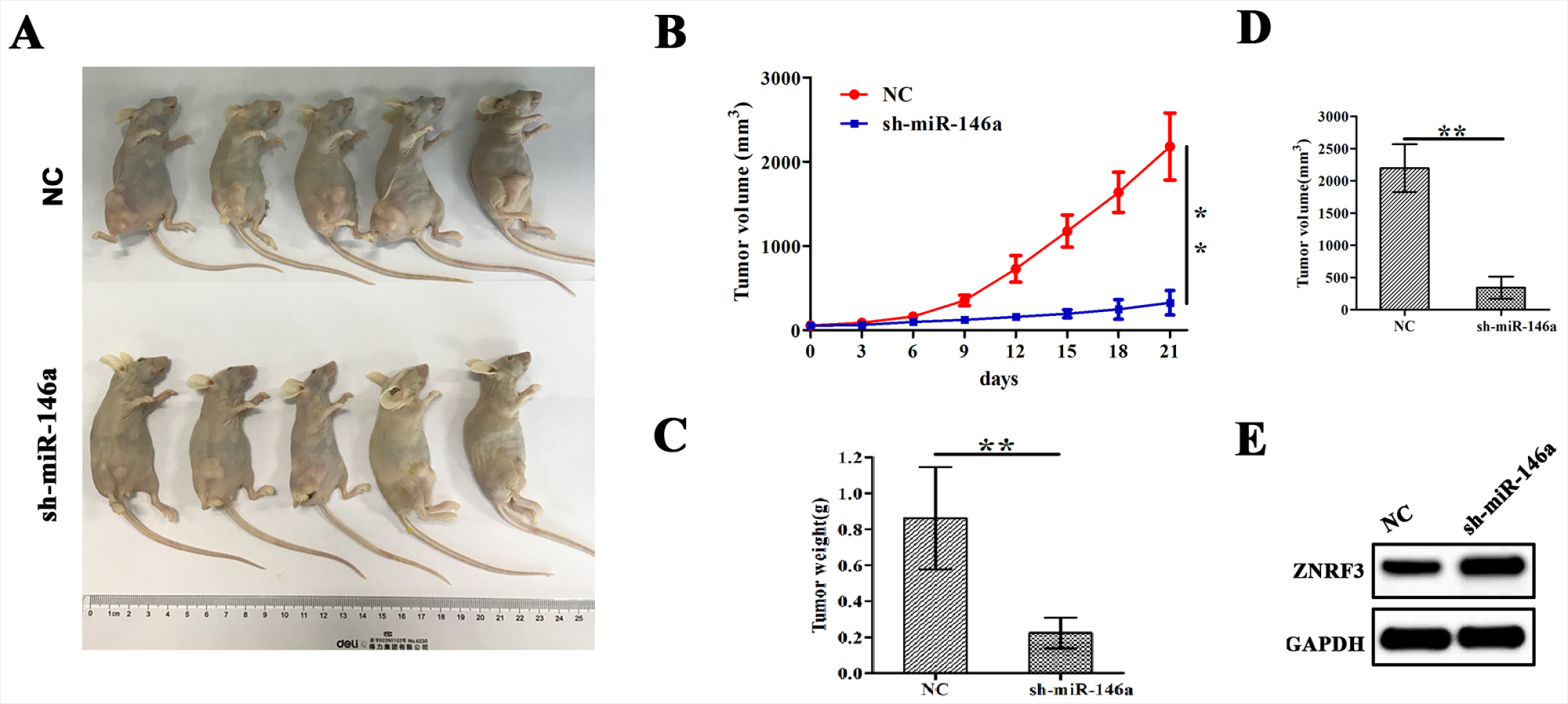
Knockdown of miR-146a inhibited U-2OS xenograft tumor growth **(A)** Schematic representation of the subcutaneous xenograft tumor size between sh-miR-146a and NC groups. **(B)** The effects of miR-146a knockdown on tumor proliferation activity of U-2OS xenograft tumors. **(C, D)** The effects of miR-146a knockdown on the average weight and volumes in xenograft tumors on the 21st day. **(E)** Western blotting analysis of the effect of miR-146a knockdown on ZNRF3 expression. **P<0.01.

Having confirmed the proliferation-promoting role of miR-146a in OS cells *in vitro,* we further verified the effects of miR-146a on OS cell growth *in vivo*. A subcutaneous U-2OS tumor model was esbablished to assess the growth activity of xenograft tumors affected by miR-146a. During the 3 weeks’ investigation, the tumor length and width of the xenograft tumors was examined every other day. We found that the proliferation rates of tumors were significantly decreased by sh-miR-146a (Figure 7A and 7B). The average weight and volumes in sh-miR-146a group were lowered compared with those in NC group (Figure 7C and 7D). Further, western blotting analysis showed that knockdown of miR-146a downregulated the expression of ZNRF3 in xenograft tumor tissues (Figure 7E).

## DISCUSSION

Accumulating evidence shows that miR-146a is implicated in cell growth and metastasis in various cancers, of which the dysregulation of miR-146a is associated with pathological staging, poor differentiation and distant metastasis, acting as an independent prognostic element for overall survival in gastric cancer [25, 26] and lung cancer [27]. In addition, miR-146a polymorphism rs2910164 is linked to the risk of esophageal cancer and colorectal cancers [28, 29]. In this study, we analyzed the correlation of miR-146a with clinicopathologic characteristics and prognosis of OS patients, which indicated that increased expression of miR-146a was positively linked to tumor size and OS recurrence, but Kaplan-Meier analysis indicated that miR-146a was not an independent prognostic factor for overall survival and recurrence for OS patients, unveiling that miR-146a might be a potential biomarker for OS patients.

Functionally, in terms of the diversity of tissue expressions and the difference in target gene function, imiR-146a can act as an oncogene [12-14] or a tumor suppressor [21-24] in a variety of malignancies. miR-146a inhibits cell invasion in pancreatic cancer via inhibition of EGFR and NF-kB pathways [30], weakens the metastatic potential in breast cancer [31] and reduces epithelial-mesenchymal transition of esophageal carcinoma [32]. But, the function of miR-146a in human OS tissues is elusive. To confirm its role in human OS, our results showed that knockdown of miR-146a inhibited growth and colony formation *in vitro* and *in vivo*, revealing the tumor-promoting role of miR-146a in OS cells.

**Figure 8:**
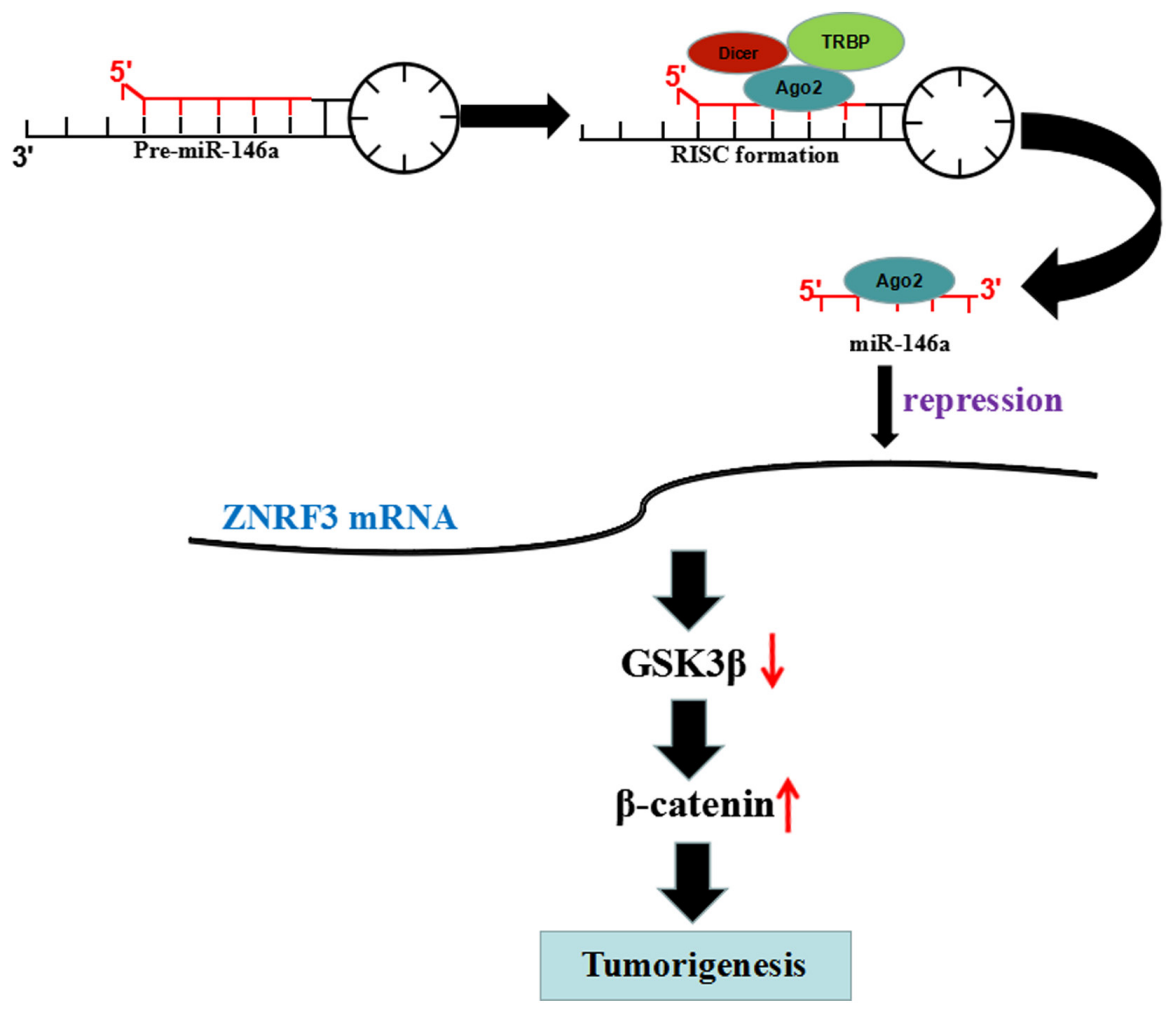
miR-146a promoted OS tumorigenicity via regulation of ZNRF3/GSK-3β/β-catenin signaling pathway miR-146 reduced the transcriptional level of ZNFR3 gene by binding to its 3’ UTR, upregulated the expression of p-GSK-3β and β-catenin, but downregulated GSK-3β expression, thereby leading to the tumorigenesis of OS.

ZNRF3 as a member of E3 ubiquitin ligases family is downregulated in gastric cancer [33] and papillary thyroid carcinoma [34] and colorectal cancer [35], and inhibits cell growth and invasion and induces apoptosis via regulation of the Wnt/β-catenin pathway [33, 36]. MiR-93 promotes growth of lung cancer cells via ZNRF3/Wnt/β-catenin axis [37], and ZNRF3 suppresses the tumorigenesis and metastasis of nasopharyngeal carcinoma cells by inhibition of Wnt/β-catenin signaling [38]. Intriguingly, we also found that ZNRF3 was a direct target of miR-146a in OS cells and was negatively correlated with miR-146a expression in OS tissues; Overexpression of ZNRF3 repressed cell growth and rescued the tumor-promoting role of miR-146a via inhibition of GSK-3β/β-catenin signaling, indicating that miR-146a might promote OS tumorigenicity via targeting ZNRF3/GSK-3β/β-catenin signaling (Figure 8).

Taken together, our study demonstrates that miR-146a is highly expressed in OS tissues and has the positive correlation with tumor size and high recurrence of OS patients. Restoration of miR-146a expression promotes OS cell growth via targeting ZNRF3/GSK-3β/β-catenin pathway, and represents a promising biomarker for OS patients.

## MATERIALS AND METHODS

### Materials

OS cell lines (U-2OS, Saos-2, MG-63, SW-1353, HOS, 143B) used in this study were from Laboratory of Orthopaedic Surgery of Renji Hospital. The materials and reagents used in this study were summarized in Supplementary Table 4.

### Cell culture and transfection

Cell resuscitation and frozen are manipulated according to the standard process. OS cells were cultured with DMEM medium added with 10% FBS and 1% penicillin (100U/ml) and streptomycin (100μg/ml) in a humidified atmosphere containing 5% CO2 at 37°C. Lentivirus-mediated miR-146a or miR-146 shRNA vector construction and cell transfection were referred as previously described [39]. The transfection efficiency of miR-146 or miR-146a shRNA were determined by qPCR.

### Quantitative real-time PCR (qRT-PCR)

Total RNA exaction, reverse transcription and cDNA amplification were manipulated depending on the protocols provided by the manufacturers, of which miR-146a exaction was based on the protocols provided by miRNeasy mini kit. The primers of miR-146a, U6, ZNRF3 and GAPDH were listed in Supplementary Table 5. Data were analysed using the comparative Ct method (2^-∆∆Ct^). Three separate experiments were performed for each clone.

### Western blot analysis

OS cells and xenograft tumor tissue cells were harvested and extracted using lysis buffer (Tris-HCl, SDS, Mercaptoethanol, Glycerol)., and the experimental protocols for western blot analysis was performed as previously reported [40].

### Cell viability assay

OS cells transfected with miR-146a, miR-146a shRNA and ZNRF3 were incubated in 96-well-plates at a density of 2×103 cells. Cells were pretreated with 10μl of MTT dye each day. After incubation for 4 h, 100μl of DMSO were added into the cells for 15 min. The color reaction was measured at 570 nm using an Enzyme Immunoassay Analyzer (Bio-Rad, Hercules, CA).

### Colony formation assay

OS cells transfected with miR-146a, miR-146a shRNA and ZNRF3 were trypsinized and reseeded into 6-well plate. The number of cell colonies in control and treatment groups were counted after a week and were stained with crystal violet.

### Dual-luciferase reporter assay

OS cells were cultured in 24-well plates. Gene report vector containing wild type 3′-UTR or mutated 3′-UTR of ZNRF3 target gene was co-transfected with negative control or miR-146a into the OS cells. The detained manufacturer’s instructions were referred to previously published [41].

### Animal experiments

Six-week-old female immune-deficient nude mice were fed in Animal Institute of Chinese Academy of Sciences based on the regulations and internal biosafety and bioethics guidelines of Renji Hospital, Shanghai Jiao Tong University. Mice were injected subcutaneously with 1×10^6^ U-2OS cells stably transfected with sh-miR-146a or control cells. The length and width of the Xenograft tumors was measured with a caliper every other day.

### Statistical analysis

SPSS 21.0 was applied for the statistical analysis of the values, which were recorded as the mean ±SEM. Paired or independent t test was used to analyse the differential change in each group. Statistical significance was set at *P* < 0.05.

## SUPPLEMENTARY MATERIALS TABLES


