# Supplementary material for: miR-146a promotes growth of osteosarcoma cells by targeting ZNRF3/GSK-3β/β-catenin signaling pathway
Source: Oncotarget. 2017 Jul 19;8(43):74276–86. doi: 10.18632/oncotarget.19395 (PMC5650339; doi:10.18632/oncotarget.19395)
Supplement: Supplementary file 1 [file oncotarget-08-74276-s001.pdf]

miR-146a promotes growth of osteosarcoma cells by targeting ZNRF3/GSK-3β/β-catenin signaling pathway

SUPPLEMENTARY MATERIALS

Supplementary Table 1: Univariate and multivariate analyses of factors associated with overall survival

Factors	Overall survival			
		Multivariate		
	Univariate *P*	HR	95% CI	*P* value
Age (years) (≥20 vs. <20)	0.085			NA
Gender (male vs. female)	0.863			NA
Tumor size (cm) (≥3 vs. <3)	0.250			NA
Lymph node infiltration (Yes vs. No)	0.152			NA
Distant metastasis (Yes vs. No)	0.606			NA
miR-146a expression (high vs. low)	0.781			NA

Supplementary Table 2: Univariate and multivariate analyses of factors associated with recurrence

Factors	Recurrence			
		Multivariate		
	Univariate *P*	HR	95% CI	*P* value
Age (years) (≥20 vs. <20)	0.504			NA
Gender (male vs. female)	0.343			NA
Tumor size (cm) (≥3vs. <3)	0.903			NA
Lymph node infiltration (Yes vs. No)	0.339			NA
Distant metastasis (Yes vs. No)	<0.0001	3.179	1.741-5.805	<0.0001
miR-146a expression (high vs. low)	0.217	1.070	0.950-1.205	0.266

Supplementary Table 3: Target genes of miR-146a-5p in cancer tissues

Name	Gene name	Position	Target Scan sites	Pic tar sites	RNA22 sites	PITA sites	miR and aSites	Program num	Cancer num
**hsa-miR-146a-5p**	**ZNRF3**	**chr22:29453230-29453236[+]**	**26[5]**	**0[0]**	**50[6]**	**0[0]**	**50[6]**	**3**	**8**
hsa-miR-146a-5p	ELAVL1	chr19:8027280-8027286[-]	55[5]	0[0]	0[0]	55[5]	55[5]	3	5
hsa-miR-146a-5p	ELF2	chr4:139979700-139979721[-]	0[0]	0[0]	2623[12]	2623[12]	2623[12]	3	7
hsa-miR-146a-5p	ZNF512B	chr20:62588308-62588315[-]	85[5]	85[5]	0[0]	0[0]	85[5]	3	6
hsa-miR-146a-5p	KCTD15	chr19:34304347-34304354[+]	783[12]	0[0]	391[12]	391[12]	391[12]	4	6
hsa-miR-146a-5p	IRAK1	chrX:153277247-153277254[-]	55[3]	0[0]	0[0]	55[3]	81[5]	3	5

Supplementary Table 4: List of materials and regents

Materials and reagents	Company name	Origin and country
miR-146a vector	Genechem	Shanghai, PR, China
miR-146a shRNA vector	Genechem	Shanghai, PR, China
ZNRF3 vector	Genechem	Shanghai, PR, China
negative control vector	Genechem	Shanghai, PR, China
virion-packaging elements	Genechem	Shanghai, PR, China
All antibodies	Cell Signaling Technologies	Beverly, MA, USA
Dulbecco’s Modified Eagle medium (DMEM)	Thermo Fisher Scientific Inc	Waltham, MA, USA
fetal bovine serum (FBS)	Thermo Fisher Scientific Inc	Waltham, MA, USA
3-(4,5)-dimethylthiahiazo (-z-yl)-3,5-di-phenytetrazoliumromide (MTT)	Dingguo biology	Shanghai, PR, China
TRIzol Reagent	Invitrogen	Carlsbad, CA, USA
Lipofectamine 2000	Invitrogen	Carlsbad, CA, USA
M-MLV Reverse Transcriptase	Promega	Madison, WI, USA
SYBR Green Master Mixture	Takara	Otsu, Japan
RNase A	KeyGEN biology	Nanjing, PR, China
ECL-PLUS/Kit	GE Healthcare	Piscataway, NJ, USA
miRNeasy mini kit	invitrogen	Carlsbad, CA, USA

Supplementary Table 5: List of primers

Gene	Sense primer	Antisense primer
miR-146a	5’-GCGAGGTCAAGTCACTAGTGGT-3’	5’-CGAGAAGCTTGCATCACCAGAGAACG-3’
U6	5’-CTCGCTTCGGCAGCACA-3’	5’-AACGCTTCACGAATTTGCGT-3’
ZNRF3	5’-CATCGTCAACAAGCAGAAAGTG-3’	5’- GGAGACCACG ACGAAGAAAG-3’
GAPDH	5’-GCACCGTCAAGGCTGAGAAC-3’	5’-TGGTGAAGACGCCAGTGGA-3’
